# Evaluating community deliberations about health research priorities

**DOI:** 10.1111/hex.12931

**Published:** 2019-06-28

**Authors:** Susan Dorr Goold, Marion Danis, Julia Abelson, Michelle Gornick, Lisa Szymecko, C. Daniel Myers, Zachary Rowe, Hyungjin Myra Kim, Cengiz Salman

**Affiliations:** ^1^ Department of Internal Medicine, Division of General Medicine, Institute for Healthcare Policy and Innovation Center for Bioethics and Social Sciences in Medicine Ann Arbor Michigan; ^2^ Warren Magnuson Clinical Center National Institutes of Health Bethesda Maryland; ^3^ Department of Clinical Epidemiology and Biostatistics McMaster University Hamilton Ontario Canada; ^4^ Center for Bioethics and Social Sciences in Medicine University of Michigan Ann Arbor Michigan; ^5^ Department of Political Science University of Minnesota Minneapolis Minnesota; ^6^ Friends of Parkside Detroit Michigan; ^7^ Center for Statistical Computation and Research University of Michigan Ann Arbor Michigan; ^8^ Present address: Department of American Culture, College of Literature, Science and the Arts University of Michigan Ann Arbor Michigan

**Keywords:** community‐based participatory research, health priorities, research priorities, resource allocation

## Abstract

**Context:**

Engaging underrepresented communities in health research priority setting could make the scientific agenda more equitable and more responsive to their needs.

**Objective:**

Evaluate democratic deliberations engaging minority and underserved communities in setting health research priorities.

**Methods:**

Participants from underrepresented communities throughout Michigan (47 groups, n = 519) engaged in structured deliberations about health research priorities in professionally facilitated groups. We evaluated some aspects of the structure, process, and outcomes of deliberations, including representation, equality of participation, participants’ views of deliberations, and the impact of group deliberations on individual participants’ knowledge, attitudes, and points of view. Follow‐up interviews elicited richer descriptions of these and also explored later effects on deliberators.

**Results:**

Deliberators (age 18‐88 years) overrepresented minority groups. Participation in discussions was well distributed. Deliberators improved their knowledge about disparities, but not about health research. Participants, on average, supported using their group's decision to inform decision makers and would trust a process like this to inform funding decisions. Views of deliberations were the strongest predictor of these outcomes. Follow‐up interviews revealed deliberators were particularly struck by their experience hearing and understanding other points of view, sometimes surprised at the group's ability to reach agreement, and occasionally activated to volunteer or advocate.

**Conclusions:**

Deliberations using a structured group exercise to engage minority and underserved community members in setting health research priorities met some important criteria for a fair, credible process that could inform policy. Deliberations appeared to change some opinions, improved some knowledge, and were judged by participants worth using to inform policymakers.

## 1 INTRODUCTION

A major contributor to health disparities is the relative lack of resources—including the resources of science—allocated to address the health problems of those with disproportionately greater needs.^1^, ^2^ While health research priorities are often shaped by scientists, clinicians, advocacy groups and the private sector, the allocation of scarce resources for health research requires explicit attention to both justice *and* science.^3^, ^4^ Engaging and involving underrepresented communities in research priority setting could make the scientific research agenda more equitable, and more responsive to their needs and values.^5^, ^6^, ^7^

Academics, funders and governments increasingly strive to engage communities not merely as subjects of research, but as partners in the setting of priorities for health research.^8^ The Council of Public Representatives, charged with advising the NIH Director on research priorities, recommended educating and involving the public, “where they live,”^3^, ^9^, ^10^, ^11^, ^12^ yet *how* to engage communities in research priority setting remains a challenge. Those seeking to involve the public in setting priorities for limited resources sometimes use a deliberative approach, which aims for collective, informed problem‐solving about a policy problem.^13^ Trade‐offs between different areas of spending can be difficult policy topics.^10^, ^14^ and those that wrestle with core values, or that pit money against health, can be particularly difficult. Research allocation decisions may not seem salient to many non‐experts, and discussions about health research priorities can be complex and technical, so members of the public may not feel competent to contribute. Given the challenges of deliberations on complex and value‐laden topics, attention to the quality of deliberation is essential.

In this paper, we evaluate the use of a deliberative exercise, CHAT (CHoosing All Together), to facilitate deliberation about health research priorities constrained by limited resources.^†^ CHAT was originally developed as a “serious game” for deliberations about the design of health insurance plans^15^ that aims to promote informed, reasoned dialogue about allocation decisions among ordinary persons.^16^ It has been used to examine health‐care priorities in a number of different settings in the USA and other countries, engaging a wide range of individuals and communities.^15^, ^17^, ^18^, ^19^, ^20^, ^21^, ^22^, ^23^, ^24^ A number of studies have concluded, in these settings, that CHAT facilitates high‐quality deliberation, changes individual preferences and opinions and increases knowledge.^15^, ^18^, ^20^ There is some evidence that CHAT leads participants to take a more public‐spirited view of resource allocation decisions; for example, a 2004 study found that participants in CHAT were willing to give up some benefit coverage to increase coverage of the uninsured.^20^


† We describe the priorities for health research spending selected by participants using this exercise elsewhere.^35^, ^47^
Setting priorities for health insurance or health care, while complex and value‐laden, can be viewed by most people as potentially relevant to their lives, whereas priorities for health research could seem more remote. Whether CHAT can produce high‐quality deliberation on this complex topic, further from the day‐to‐day experience of most members of the public, is unknown. Here we report an evaluation of deliberations about such prioritization decisions using CHAT to facilitate deliberation about the allocation of health research dollars in minority and medically underserved communities.

We evaluate CHAT deliberations using a framework that examines the formal *structure* of deliberation (how it is organized), the *process* of deliberation (how it transpires) and the *outcomes* produced (Table 1).^25^, ^26^, ^27^, ^28^, ^29^ While the goal of deliberation could be construed as “better” decisions, or outcomes, much of the normative value of deliberation comes from its promise of offering a fair process of discussion and decision making, independent of the decisions actually reached. Theories of deliberative democracy, despite important differences, share an emphasis on a process in which political actors listen to each other with openness and respect, provide reasons and justifications for their opinions, remain open to changing their points of view and consider the common good.^30^, ^31^ Structural elements can include information and choices, materials, tasks, sampling and group composition.^32^ Examples of procedural aspects of quality include respectful treatment, civility and reason‐giving. Outcomes can include changes in participants’ knowledge or opinions, decisions made and participants’ views of the group decision, including trust in decision makers.^30^, ^33^ These domains may interrelate; for instance, representation (one element of structure) could influence the quality of deliberations (process) and/or changes in the point of view of participants (outcome). Evaluation of the quality of deliberative approaches, despite its importance, is a nascent field of study.^26^, ^27^

**Table 1:** Analytical framework for evaluating deliberations using CHAT

Elements of evaluation	Criteria	Data Sources
Structures	Information and Choices Materials Tasks and exercises Sampling and group composition	Survey items measuring deliberators’: o Demographics o Views of the quality of information and choices available
Processes	Respectful treatment Civility Reason‐giving	Survey items measuring deliberators’: o Perceptions of being treated with respect o Opportunities to present their points of view o Views of group discussion o Support for using their group's decision to inform decision makers o Trust in a process like this to inform decision makers Documentation of deliberators’ participation in group discussion
Outcomes	Changes in knowledge or opinion Individual and group decisions Participant views of group decision	Survey items measuring deliberators’: o Knowledge about health research, disparities, and social determinants of health o Trust in medical researchers o Willingness to participate in research o Likelihood of becoming a participant in health research in the future o Perceived and desired input on setting research priorities Follow‐up interviews with participants Individual and group health research priorities selected using CHAT

We evaluated the structure, process and outcomes of deliberations from the perspective of deliberators themselves, how they viewed the process, whether and how knowledge and attitudes changed, and what they thought about using such a process to inform decision makers.

## 2 METHODS

To adapt CHAT to the unique needs and objectives of research priority setting with minority and underserved communities, we utilized a participatory process, led by a Steering Committee comprised of a majority of community leaders and several leaders of research institutions, that engaged community partners in all phases of the project.^34^ Adaptation was informed by documents and interviews with funders, research institutions, clinicians and community members.

CHAT content was designed to be credible and comprehensible to a lay audience. Final content (which included definitions and explanations of a number of scientific terms) had a Flesch‐Kincaid readability score of 55 and was written at an 8th grade reading level (See Table S1). All content was available in both English and Spanish.

**Figure 1:**
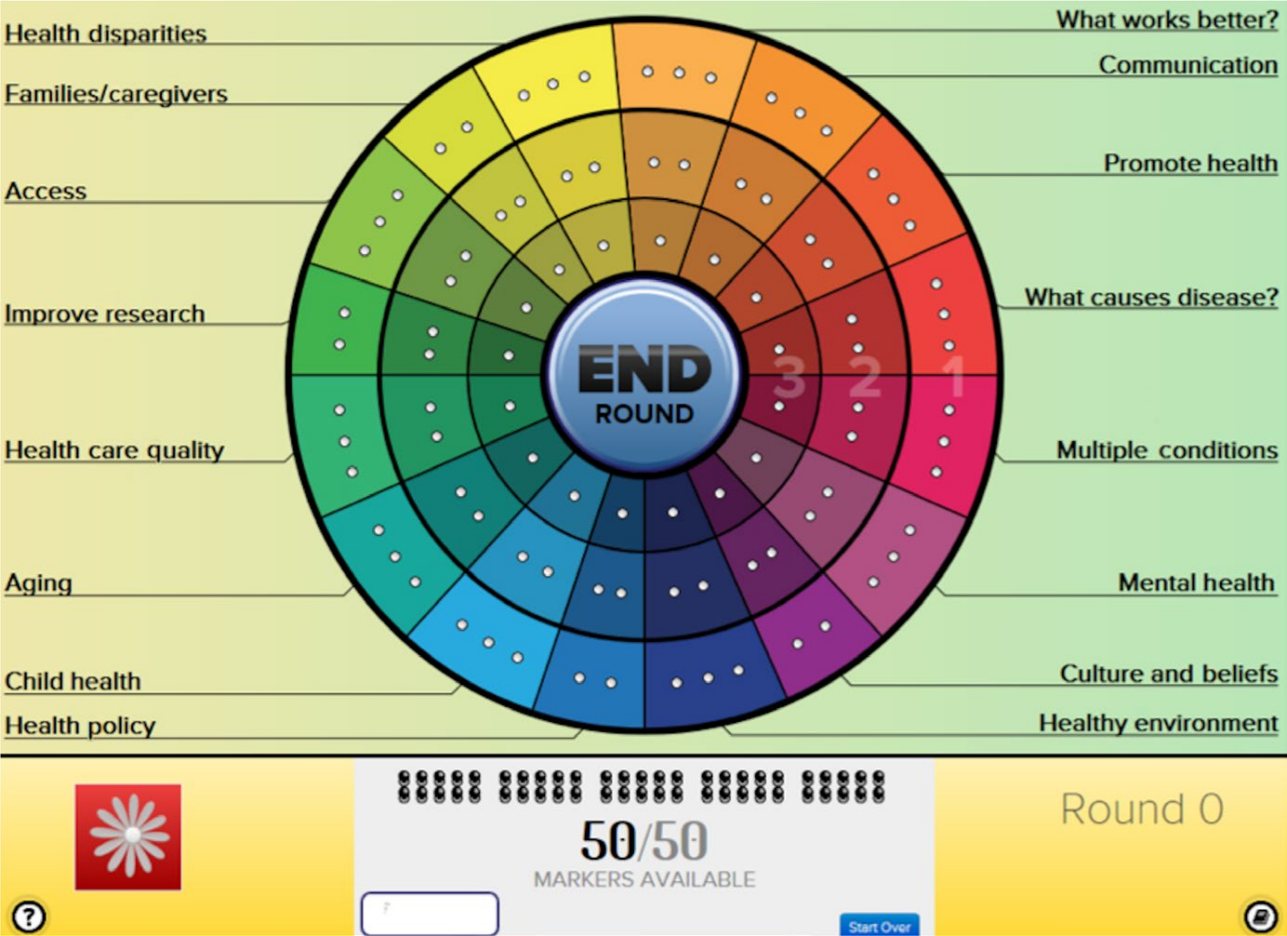
Screenshot of the CHAT wheel. Each wedge represents a category of research. Descriptions of each category and what is provided by the different levels of investment (level 1 = outer ring, level 2 = outer + middle rings, level 3 = outer + middle + inner rings) appear with clicking on a wedge. For each wedge, participants can choose not to allocate any of their 50 available markers, or they can use the number of markers needed for level 1, 2 or 3

Since participants were laypersons with varying levels of baseline knowledge, sessions began with a brief video about health research goals, methods, costs, funders and uses, and introduced deliberators to their task. Tablet devices displaying the CHAT exercise presented participants with an interactive game board (Figure 1) with spending options depicted as wedges of a circle. Each of the 16 wedges represented a category of health research spending, and each wedge had different levels of spending (including the option of no spending at all); higher levels (towards the centre of the wheel) present a greater investment in that type of research. Categories and levels are described in Table S1 and previously published work.^35^

Participants chose a level of funding for each category by allocating markers required for the particular level. However, participants were given a limited number of markers (50 markers for 92 open spaces) so choosing high levels of funding in one category required lower or no funding in another. Participants allocated their markers in four rounds. In the first round, participants set priorities as individuals; in the second round, they set priorities in small groups of 2‐4; in the third round, they set priorities with the entire group (up to 15); and in the fourth round, they set priorities again as individuals. After rounds 1 and 2, the group heard and discussed scenarios (“events”) that illustrated the consequences of their choices. In round 3, deliberators were asked to articulate reasons for their priorities. In all rounds, trained facilitators asked deliberators to make fair decisions on behalf of fellow community members. Participants learned from other members of the group, the illustrative events and embedded resources.

### 2.1 Sampling and recruitment

We aimed to recruit equal numbers of men and women, and to have disproportionate representation of minority and low‐income residents, since these perspectives tend to be underrepresented in decisions about health research priorities.^10^, ^11^, ^12^ Purposive sampling targeted minority and medically underserved communities throughout the state of Michigan.^36^ Recruitment involved a variety of local advertising (newspapers, craigslist, radio) in English and Spanish, posting and distribution of flyers through community‐based organizations, the website UMHealthResearch.org and occasionally personal contacts. Volunteers were excluded if they reported currently working in health care or health research, or if they were under 18 years of age. We convened 47 focus groups of 4‐15 participants across the state of Michigan (Total n = 519, see Figure S1) in locations familiar to and convenient for participants to maximize open and frank dialogue. Two groups were conducted in Spanish.

### 2.2 Data collection

Given the complexity of public deliberations about health research priorities, we aimed to evaluate multiple aspects of the deliberation structure, process and outcomes (Table 1). Data sources included pre‐ and post‐deliberation surveys, research staff observations of deliberations, priorities selected by individuals before and after group deliberations (previously reported),^35^ and follow‐up interviews with one participant from each group a year after the deliberations were conducted. Missing data for survey responses ranged from 0% to 7%.

#### 2.2.1 Structure

We measured representativeness using participants’ self‐reported demographic characteristics. Given our goal to engage minority and underserved communities, racial and ethnic minority and lower‐income individuals needed to be disproportionately included. Poverty level was calculated using the upper portion of self‐reported income range and the number of individuals living in their household. This approach is a conservative estimate of the number of participants living under the federal poverty level. We included six questions in the post‐deliberation survey to measure deliberators’ views of the quality of information and the choices available.^15^, ^25^, ^26^, ^27^, ^28^, ^29^

#### 2.2.2 Process

We measured multiple elements of the deliberative process. Thirteen items in post‐deliberation surveys measured various dimensions of deliberative quality perceived by participants, including respectful treatment, opportunity to contribute their point of view and their views of the kinds of arguments offered in deliberation.^15^, ^24^, ^26^, ^27^, ^28^, ^29^ Mean responses are reported on a 0‐4 Likert scale from Strongly Disagree to Strongly Agree, with some items reverse‐coded so that higher scores always indicate higher deliberative quality. Post‐deliberation surveys also included two items measuring whether deliberators supported using their group's decision to inform decision makers, and their trust in a process like this to inform decision makers; while not direct measures of process quality, we expect deliberators to support or trust processes like this to inform policy only if they view them as credible, legitimate and fair.

In addition to survey measures, the distribution of contributions by deliberators was measured by members of the research team at 41 of the 47 sessions; at six sessions, staff was insufficient to allow complete recording of participation. Using a diagram of the deliberators, they hand‐recorded the number of times each person spoke, a more accurate way to capture this information than transcription. We used this information to assess and compare equality of participation between groups using a standard metric for market concentration, the Herfindahl‐Hirschman Index (HHI),^37^ which measures the degree to which one or a few actors dominate any setting. Here, we used the HHI to measure the degree to which discussion was dominated by one or a few people.

#### 2.2.3 Outcomes

To measure the impact of participation on deliberators’ knowledge about research and health disparities, we compared their responses on pre‐ and post‐deliberation surveys. Knowledge of health research was measured using two new instruments, after a search revealed no validated measures available. One instrument presented three vignettes and asked participants whether or not the vignette was research. The other instrument presented statements about research and research funding, and asked respondents to rate them true or false, for example “Results from research need to be repeated to make sure they are believable,” and “The federal government funds a great deal of health research.” Both measures of knowledge about research were cognitively pretested. Knowledge of health disparities was tested using a single item based on the standard definition:

Which of the following do you think is the best way to define “health disparities?”

1. Health disparities are differences in the health‐care people receive.
2. [correct] Health disparities are particular types of health difference closely linked with social, economic and/or environmental disadvantage.
3. Health disparities are health differences between racial and ethnic groups.
4. I don't know.

Post‐deliberation surveys also measured trust in medical researchers,^38^ willingness to participate in research, likelihood of future participation in health research and perceived and desired input on setting research priorities.

One year after the final group deliberation, we randomly selected, from those who agreed to be re‐contacted (86% of participants), one participant from each CHAT group. Semi‐structured interviews, conducted by phone, asked about their recall of CHAT sessions, and about the impact of participation on them or others, with appropriate probes (eg whether they sought information about scientific research, took part in any action related to science or science policy, discussed health research priorities with others) (Appendix Interview Guide). Due to the nature of semi‐structured interviews and time constraints of interviewees, not every question was asked of every interviewee. Interviews were recorded and transcribed verbatim.

### 2.3 Analysis

Descriptive results include means for scale scores (eg views of information) and individual survey items (eg willingness to participate in future research). Proportions describe some demographics and correct responses for knowledge items.

We analysed all questions measuring deliberators’ perceptions of the quality of the deliberative process and structure using principal components analysis. As expected, items loaded onto different scales depending on whether they measured the sufficiency of information and choices, or the quality of deliberation itself. Items measuring the quality of information and choices loaded onto two separate scales depending on whether they were phrased positively or negatively; we label these “Sufficient Information and Choices” and “Insufficient Information and Choices.” We expected items measuring the quality of deliberation to load onto separate factors for different elements of deliberative quality (eg mutual respect, quality of argumentation). However, the PCA results strongly suggested that these items formed a single factor, which we label “Views of Deliberation.” Factor analysis revealed similar domains.

We used multilevel regression to examine relationships between participants’ demographic characteristics (age, gender, race, ethnicity, education, income and rural residence), their views of the deliberation and its information and choices, and their overall trust in or support for using this process to inform policy. Changes from pre‐ to post‐deliberation were assessed adjusting for within‐participant responses nested within‐CHAT group using multilevel regression models for knowledge of health disparities (percentage correct), and using multilevel logistic regression models for dichotomized responses of perceived and desired input on research priorities (some or a great deal vs a little or none at all), likelihood of participation in research (somewhat likely or very likely vs somewhat unlikely or very unlikely) and willingness to take part in research (somewhat willing or very willing vs somewhat unwilling or very unwilling). For calculating percentage correct knowledge responses, if at least one item within the set of knowledge questions is answered, then a missing response is considered an incorrect response.

To examine the distribution of participation in deliberations, we calculated the Herfindahl‐Hirschman index (HHI) for each group:

$$\text{HHI}=\sum_{i=1}^{n}{\left(\frac{X_{i}}{X}\right)}^{2}$$

where *X* represents the total number of contributions to the deliberation, *X_i_* represents the number of contributions of an individual *i* (so *X_i_/X* is the share of contributions by individual *i*), and *n* is the total number of individuals in a deliberating group. To allow comparisons between groups of different sizes, we calculated the normalized HHI^39^:

$${\text{HHI}}^{N}=\frac{\text{HHI}-1/n}{1-1/n}.$$

A normalized HHI is zero when there is complete equality of participation and 1 when there is a complete concentration of participation.

Analysis of follow‐up interviews with CHAT participants was descriptive rather than interpretive guided by the method of qualitative description.^40^ Transcripts were coded by question, and two or more investigators developed codes (labels) for responses. For example, when asked “What was it [playing CHAT] like?” the term “interesting” arose often (21 of 36 interviewees); coders then categorized the different ways or reasons participants found it interesting, for example 11 of 21 mentioned hearing other points of view.

This study was reviewed by the University of Michigan IRBMED and deemed to be exempt from review. Nonetheless, at the beginning of each session, the facilitator explained that it was not possible to assure the privacy of all information given the presence of others in the room, that the research team would not collect or retain any identifying information, and asked participants to respect the privacy of other members of the group.

## 3 RESULTS

### 3.1 Structure

Deliberators ranged from 18 to 88 years old, with 20% over 65 (Table 2). About two‐thirds were women and about one‐third resided in a rural area. About half self‐identified as White, 31% Black/African American, 7% Hispanic, 6% Native American and 4% Arab American, Arab or Chaldean. Most participants (63%) had incomes less than $35,000; at least 157 (32.6%) were under the federal poverty level. About half (48.0%) reported very good or excellent health. Compared with the population of Michigan, our sample overrepresented minority and low‐income residents.

**Table 2:** Participant characteristics

Participant characteristics	N (%) Except as noted
Female	351 (67.6)
Age in y (n = 509), mean (SD, range)	48.3 (17.6, 18‐88)
Self‐identified Race (n = 505)	
White	252 (49.9)
Black or African American	158 (31.3)
Other, including multiracial	95 (18.8)
Native American	32 (6.1)
Arab American	23 (4.4)
Hispanic (n = 481)	35 (7.3)
Education (n = 510)	
High school/GED or Less	140 (27.5)
Some college	192 (37.7)
Bachelor's degree or more	178 (34.9)
Region (n = 519)	
South East	230 (44.3)
South West	102 (19.7)
North	109 (21.0)
Upper	58 (11.2)
Thumb	20 (3.9)
Urbanity (n = 494)	
Urban	298 (60.3)
Suburban	25 (5.1)
Rural	171 (34.6)
Income (n = 490)	
Less than $15 000	165 (33.7)
$15 000 to $34 999	144 (29.4)
$35 000 or more	181 (36.9)
No. of people in household (n = 503), mean (SD; range)	2.7 (1.5; 1‐9)
At or below 100% federal poverty level (n = 481)	157 (32.6)
At or below 200% federal poverty level (n = 482)	257 (53.3)
Living alone (n = 502)	118 (23.5)
Perceived health status (n = 511)	
Fair or poor	87 (17.0)
Good	179 (35.0)
Very good or excellent	245 (48.0)
Work or worked in health care or health research (n = 510)	193 (37.8)
Currently work in health care or health research	77 (15.1)
Health care	72 (13.7)
Health research	3 (0.6)
Missing	3 (0.6)

Mean item and scale scores (Table 3) describe generally favourable views of the information and choices provided. Those with a high school education or less had lower scores on the sufficient views of information and choices and views of discussion scales (Table 4). Those with higher incomes rated the sufficiency of information and choices more highly.

**Table 3:** Participants’ views of information, choices and deliberation

	Mean (SD, Range)
Sufficient Information and Choices scale^a^	2.9 (0.7, 0.0‐4.0)
The information given to us was believable.	3.0 (0.9, 0.0‐4.0)
The choices offered in the exercise were realistic.	2.9 (0.9, 0.0‐4.0)
The choices in the exercise included the choices I could have wanted.	2.8 (0.8, 0.0‐4.0)
There was a wide selection of choices.	2.9 (0.8, 0.0‐4.0)
Insufficient Information and Choices scale^b^	2.3 (0.8, 0.0‐4.0)
We did not have enough information to make good decisions. (−)	2.5 (1.0, 0.0‐4.0)
There were choices I would have liked to have seen but didn't. (−)	2.0 (1.0, 0.0‐4.0)
Views of Deliberation^c^	2.8 (0.5, 0.9‐4.0)
A few people dominated the discussions (−)	2.3 (1.1, 0.0‐4.0)
The way in which the group reached its decision was not fair (−)	3.0 (0.9, 0.0‐4.0)
The discussions were superficial (−)	2.8 (0.9, 0.0‐4.0)
There was too little time to discuss (−)	2.5 (1.0, 0.0‐4.0)
People in the group argued by referring to what would be best for themselves (−)	2.4 (1.1, 0.0‐4.0)
Our discussion included responding to each others' arguments	2.8 (0.8, 0.0‐4.0)
I gained understanding of the arguments that opposed my own	2.9 (0.7, 0.0‐4.0)
My views were considered and taken into account	3.1 (0.7, 0.0‐4.0)
I had lots of chances to share my views	3.1 (0.7, 0.0‐4.0)
The participants in the group argued by referring to what would be best and most fair for all people	2.6 (1.0, 0.0‐4.0)
All positions were considered with equal respect	3.1 (0.7, 0.0‐4.0)
The arguments of the other participants were useful in forming my own position	3.0 (0.7, 0.0‐4.0)
During the exercise, I was treated with respect	3.4 (0.6, 0.0‐4.0)
I would support using our group's decision to inform decision makers.	3.1 (0.8, 0.0‐4.0)
I would trust a process like this to inform funding decisions.	3.0 (0.8, 0.0‐4.0)

Note

(−) Denotes reverse‐scored items.

a Mean of 4 items; each 5‐point item can range from 0 to 4. Cronbach's α = 0.81.

b Mean of 2 items; each 5‐point item can range from 0 to 4. Cronbach's α = 0.43.

c Mean of 13 items; each 5‐point item can range from 0 to 4. Cronbach's α = 0.80.

**Table 4:** Predictors of overall support for or trust in deliberation process

Dependent variables →	“I would trust a process like this to inform funding decisions”	“I would support using our group's decision to inform decision makers”	Sufficient info & choices	Insufficient info & choices	Views of Deliberation
Predictors	Beta [95% CI]	Beta [95% CI]	Beta [95% CI]	Beta [95% CI]	Beta [95% CI]
Female	0.12 [−0.02, 0.26]	0.04 [−0.10, 0.19]	0.14* [.01, 0.27]	0.22** [.06, 0.39]	0.07 [−0.02, 0.16]
Age in y	0.002 [−0.002, 0.006]	0.003 [−0.001, 0.008]	0.001 [−0.003, 0.004]	‐0.001 [−0.005, 0.004]	0.002 [−0.001, 0.005]
Race (ref: white)					
Black	0.15 [−0.03, 0.34]	0.01 [−0.19, 0.21]	0.05 [−0.12, 0.23]	0.01 [−0.21, 0.23]	0.04 [−0.09, 0.17]
Other, including multiracial	0.16 [−0.03, 0.35]	0.19 [−0.02, 0.39]	0.01 [−0.17, 0.19]	‐0.16 [−0.38, 0.07]	0.001 [−0.13, 0.13]
Hispanic	0.09 [−0.20, 0.38]	0.10 [−0.20, 0.40]	‐0.20 [−0.47, 0.06]	‐0.06 [−0.40, 0.28]	‐0.10 [−0.29, 0.09]
Education (ref: ≤high school)					
Some college	0.05 [−0.13, 0.22]	0.001 [−0.18, 0.18]	0.34*** [.18, 0.50]	‐0.03 [−0.24, 0.17]	0.17** [.05, 0.28]
College degree or more	0.04 [−0.15, 0.23]	‐0.02 [−0.22, 0.17]	0.29** [.12, 0.46]	‐0.08 [−0.30, 0.14]	0.19** [.07, 0.31]
Income (ref: <$15,000)					
$15,000 to $34,999	0.04 [−0.13, 0.22]	‐0.04 [−0.22, 0.14]	‐0.07 [−0.23, 0.09]	0.19 [−0.02, 0.39]	‐0.02 [−0.13, 0.10]
>$35,000	0.03 [−0.15, 0.21]	0.03 [−0.15, 0.21]	0.04 [−0.12, 0.20]	0.27* [.06, 0.47]	0.10 [−0.02, 0.21]
Rural residence	0.007 [−0.15, 0.17]	‐0.04 [−0.22, 0.15]	0.09 [−0.69, 0.24]	0.005 [−0.19, 0.20]	0.01 [−0.11, 0.13]
Sufficient info and choices (range 0‐4)	0.31*** [.19, 0.43]	0.30*** [.18, 0.42]			
Insufficient info and choices (range 0‐4)	‐0.04 [−0.12, 0.05]	‐0.03 [−0.12, 0.06]			
Views of deliberation (range 0‐4)	0.76*** [.59, 0.93]	0.71*** [.54, 0.88]			

Note

Multilevel regression model was used with 5‐point scale response variable and the model included random intercepts for CHAT groups to account for within‐group correlations.

* *P* < 0.05;

** *P*<0.01;

*** *P*<0.001.

### 3.2 Process

Views of deliberation were generally favourable (See Table 3). The highest rated item in the Views of Discussion scale (“During the exercise, I was treated with respect”) had a mean score of 3.4 (range, 0‐4.0). The lowest rated item in the scale (“A few people dominated the discussions”) had a mean score of 2.3. Participants, on average, agreed they would support using their group's decision to inform decision makers (Mean = 3.1 for Likert item range 0‐4) and would trust a process like this to inform funding decisions (Mean = 3.0, range 0‐4). In multivariate analyses, scores on the sufficient information and choices scale and views of deliberation scale were positively associated with support for using their group's decision to inform decision makers (beta coefficients 0.30 and 0.71, respectively, *P* < 0.001). Scores on the sufficient information and choices scale (beta coefficients 0.31 and 0.76, respectively, *P* < 0.001) and views of deliberation scale (beta coefficients 0.39 and 0.48, respectively, *P* < 0.0001) were also positively associated with trust in a process like this to inform funding decisions.

The normalized HHI, used to quantify the distribution of participation by individuals in deliberations, ranged from 0.010 to 0.097 (See Table 6). Since a normalized HHI = 0 indicates complete equality of participation, and HHI^N^=1 indicates completely monopolized dialogue, these results are consistent with relatively equal contribution frequency within each group.

### 3.3 Outcomes

Participants were more likely to correctly identify the definition of health disparities after CHAT than before (aOR = 2.2, *P* < 0.001) (Table 5). Their knowledge of health research as measured by agreement with six statements about research did not change after participation. Their proportion correct of 3 vignettes had a statistically significant although small decrease (2.9%). Participants were more likely to say they had some or a great deal of input in setting research priorities after participation, compared to before (aOR = 3.7, *P* < 0.001), and were also more likely to say they *should* have some or a great deal of input in setting research priorities (aOR = 2.3, *P* < 0.05). The proportion willing to take part in a research study, high at baseline, did not significantly change. Trust in health researchers declined slightly after participation (mean score change = −0.7, *P* < 0.001).

**Table 5:** Within‐participant Changes in knowledge of health disparity, knowledge of health research and views on health research

Health disparity definition (N^a^ = 451)	Before deliberation	After deliberation	Summary Statistics	
			OR^b^	95% CI
Health differences linked to sociodemographic disadvantages, n (%)	256 (56.8)	310 (68.7)	**2.2** b	**(1.5, 3.2)**
Health differences between racial and ethnic groups, n (%)	21 (4.7)	32 (7.1)		
Health‐care people receive, n (%)	67 (14.9)	83 (18.4)		
Don't know, n (%)	107 (23.7)	26 (5.8)		

Knowledge of health research^d^			Change^c^	95% CI
% correct of 3 vignettes (N^a^ = 464), mean (SD)	75.2 (23.7)	72.3 (24.6)	**‐2.9** **†**	**(−5.0, −0.8)**
% correct of 6 health research knowledge questions (N^a^ = 458), mean (SD)	72.8 (20.1)	72.5 (21.4)	‐0.3	(−2.1, 1.5)
% correct of 9 items (3 vignettes plus 6 questions) (N^a^ = 458), mean (SD)	73.0 (16.9)	72.0 (17.9)	‐0.01	(−0.03, 0.00)

Views about research/researchers			OR^b^	95% CI
How much input DO people like you have in setting research spending/priorities?^e^ (N^a^ = 492), n (%)	179 (36.4)	280 (56.9)	**3.7** **‡**	**(2.6, 5.3)**
How much input SHOULD people like you have in setting research spending/priorities?^e^ (N^a^ = 495), n (%)	438 (88.5)	461 (93.1)	**2.3** **†**	**(1.3, 4.0)**
Likely to become a participant in health research study^e^ (N = 457), n (%)	350 (76.6)	400 (87.5)	**5.4** **‡**	**(2.9, 10.0)**
Willing to take part in health/research study^e^ (N^a^ = 438), n (%)	409 (93.4)	417 (95.2)	1.8	(0.8, 3.9)
Trust in health researchers^f^ (N^a^ = 494), mean (SD)	2.3 (0.6)	1.7 (0.6)	**‐0.7** ‡	**(−0.7, −0.6)**

Note

All summary statistics are from hierarchical model, accounting for potential correlation of responses from within‐person nested within‐deliberation group.

Abbreviation: OR, odds ratio.

a Number of participants who responded both before and after deliberation.

b Adjusted for within‐CHAT group clustering using multilevel logistic regression when likelihood ratio test for between‐group variance was significant (*P* < 0.05).

Based on dichotomized responses to health disparity definition question as correct (“health differences linked to sociodemographic disadvantages”) vs. not correct (“health differences between racial and ethnic groups,” “health‐care people receive,” or “I don't know”)

c Mean changes are calculated as after deliberation minus before deliberation score; negative values correspond to decrease in attitudes/knowledge/trust after deliberation; adjusted for within‐CHAT group clustering using multilevel regression model when likelihood ratio test for between‐group variance was significant (*P* < 0.05).

d Three deliberation groups were not administered with knowledge questions post‐deliberation and are excluded from this analysis. If at least one item within the set of questions is answered, then missing response is considered an incorrect response.

e Collected using a 4‐point scale ranging from 0 to 3, and the dichotomized response combines “2 = some/willing/likely” or “3 = a great deal/very willing/very likely.”

f Mean of 4 items; each 5‐point item can range from 0 to 4. Scale reliability coefficient (α) is 0.44

^†^ *P* < 0.05;

^‡^ *P* < 0.001.

Of the 47 participants who were randomly selected from CHAT groups, 37 participants were interviewed, one participant refused to participate, and nine could not be reached. When asked if they remembered CHAT, about half (18/37) were able to recall aspects of their deliberations. Some mentioned specifically encountering other points of view, the need to work out differences and even changing their selections after the group deliberations:

I remember it was really difficult to prioritize because the more we got into assessing our selections, the more you could see everybody’s point of view. And it was hard to decide what…You know, how do you say that this is more important than this? It was a struggle.

I remember the process was kind of challenging like trying to prioritize what we wanted … because everyone had to kind of put away their own individual biases and just think as a group.

After the discussion, I changed the way that I answered.

Some found the experience opened their eyes to others’ life‐experiences:

I remember feeling in the meeting that there were some people there that had had a very hard load and were presenting answers or their feelings or their impressions or their thought process regarding the need at a much more personal and strong level. And I thought that that was eye‐opening.

When we did CHAT, we were still new to the town, …it was enlightening to see what the other people in our small town thought, how they felt.

Some specifically mentioned the exercise's ability to get participants to think outside of their individual perspectives:

I always knew personally what my priorities was[sic] as a person and morally, but it made me think larger than that because it made you think beyond yourself and beyond your friends and family, and it made you think for your entire state, and to think of, you know, what things matter to you and why they matter to you.

It made me a little more open to looking at different angles of an issue. That it’s not just my point of view that matters.

Participants remembered sharing their views in deliberations, even those who stayed quiet:

It…provided a way to be able to have my voice heard without actually speaking and then waiting for someone else to speak. It just provided a different type of avenue to be heard, and I liked that….I think the thing that I remembered most was that my vote counted. That meant a lot.

Others mentioned their own or community involvement in decision making:

You know, it made me aware that there’s only so much money available for research, and that…if you get a representation of the community together to make the decisions, you know, it might help the true funders to be more aware of what people want.

It actually made me feel like I was a part of…decision‐making, of helping with decision‐making in the future.

After participation, most (28 of 34) said they had spoken about their participation, typically to family or friends, sometimes to other participants and on a few occasions to community groups or organizations:

I had actually talked to my director in reference to the…to the use of the CHAT and how it helped to pull team members out, and actually did kind of minimize the voice of some of the really strong ones and allow the others to be able to speak.

Asked about any changes they attributed to participation, most did not identify any. A few mentioned being more open to and encouraging of other points of view:

I think we tried to make sure that we’re staying true to that word and be much more collaborative and work together with existing organizations in the community, and I think in my role as [redacted] I tried to be a little more disciplined in evaluating cares and concerns brought to me…whether it’s the public or staff.

Fourteen of 33 participants who were asked whether they looked for opportunities to get involved in their communities had not done so since playing CHAT. Just under a third (10 of 33) said they were already involved in their communities, and 9 said they became more involved:

I started volunteering with different mental disability groups within my community, and that wasn’t really something that I was, you know, in before, and once I came home and realized how prevalent it was just in my community, I started volunteering.

I think it motivated me. I thought I was going to retire and sit up here and read books and…take up knitting, look out at the beautiful lake and whatnot, but I have certainly gotten a lot more involved in local county and state issues.

Most interviewees had not acted on or used the project summary report they received. Some had plans to share the results, while others planned advocacy work:

No. It is on my list of things to share though with leadership here at our agency, but I haven’t acted on it yet. No.

We talk about Child Health and Mental Health and the whole stress there is right now about school closings. You know, that’s a big priority in low‐income communities… You know, making these results available to people that are out there that, you know, were looking for supporting evidence.

When asked how results could or should be used, almost all thought the results should be shared with decision makers:

…But certainly on a national level, they would be of help for politicians to know what their general public feels about certain issues.

Others thought it would be beneficial to share results with communities and thought the CHAT tool was beneficial for engaging communities in research:

I think it could be helpful if it was brought more into the communities and more people could learn more about it.

I think it’s especially important now given the climate of the political dialogue or lack of for the last 6 months.

… there’s been an adversarial relationship with the Indian Health Service because it started out as an Army program. So, something like this…. You’re open about bringing that information back to the tribe…. It’s clear that you’re working with us, not to us or on us. We need more things like that.

## 4 DISCUSSION

This paper presents an evaluation of a particular deliberative procedure engaging minority and underserved communities in deliberations with the challenging task of setting health research priorities. Consistent with our aim, we successfully overrepresented minority and low‐income residents,^41^ people who can be difficult to reach, since these perspectives tend to be underrepresented in decisions about health research priorities.

**Table 6:** Equality of participation (Herfindahl‐Hirschman Index)

Number of deliberators in group	Range of participation, %*	Normalized HHI
4	15‐40	0.047
4	14‐36	0.032
5	13‐25	0.014
5	13‐31	0.023
6	8‐28	0.044
7	6‐24	0.026
7	4‐26	0.048
8	3‐27	0.048
8	2‐27	0.056
9	6‐23	0.029
9	6‐17	0.017
9	4‐22	0.038
9	8‐16	0.010
10	0‐21	0.042
10	4‐30	0.060
10	3‐25	0.048
10	3‐23	0.039
11	0‐24	0.055
11	0‐17	0.033
11	3‐21	0.032
11	0‐21	0.045
11	0‐19	0.057
12	1‐23	0.063
12	1‐15	0.023
12	3‐18	0.018
13	0‐19	0.044
13	3‐19	0.031
13	0‐27	0.087
13	0‐15	0.034
13	1‐21	0.050
14	0‐18	0.033
14	0‐16	0.038
14	1‐19	0.051
14	1‐18	0.037
15	2‐23	0.045
15	1‐34	0.097
15	1‐19	0.044
15	1‐17	0.028
15	0‐19	0.051
15	0‐17	0.049
16	0‐12	0.019

* Proportions represent deliberators’ participation in discussion, calculated as #contributions to discussion/all contributions. For example, if Deliberator JS made 10 contributions to a discussion that included 100 total contributions, JS would have contributed 10% of the deliberation. At six of 47 sessions, sufficient staff was not present to allow complete recording of participation.

Deliberators’ views of the information and choices available were generally favourable. Those with lower educational attainment had less favourable views of the information and choices provided, which may indicate a need for additional time or resources for learning about health research and research spending. Those with lower educational attainment also had less favourable views of the discussion, although the effect was modest. Still, the experiences of those with less educational attainment warrant additional efforts in any future work to include their perspectives in deliberations about health research.

Overall, participants expressed favourable views of the deliberation. Importantly, views of the discussions, which included their perceptions of respectful treatment, equal opportunity to talk and civility, were the strongest predictor of trust in the process and support for using results to inform decision makers. This suggests that participants viewed this as a fair process for decision making, a finding consistent with similar projects.^15^, ^20^, ^23^, ^24^, ^42^

Participation was generally well distributed, even in the smallest groups, as measured by the HHI. Besides providing evidence of well‐led discussions in this project, this demonstrates the use of such an index to measure the distribution of participation in deliberations, a key element of deliberative quality. As Himmelroos has articulated, “A fair and inclusive process would subsequently be one where all participants actively take part in the exchange and evaluation of reasoned arguments.”^31^ While a combination of frequency of contributions and volume of text (or speaking time), as others have done,^27^ may permit a deeper assessment of overall contribution to discussion, the HHI provides a metric to compare and assess between groups and even between projects or events. Furthermore, even if some contributions are brief (small text volume), finding well‐distributed participation in a deliberating group, and that nearly all participants speak, provides some evidence that deliberators felt comfortable contributing. Interviews revealed the unexpected finding that some participants in CHAT felt “heard” even if they did not speak during group deliberations, and seemed to welcome having other ways to contribute their points of view. Given concerns that silence, in deliberative forums, may represent refusal to engage, or passive (even if attentive) listening,^43^ this insight about alternative ways for deliberators to engage shows some promise.

Deliberators, on average, increased their knowledge about disparities. Knowledge about health research did not seem to improve, although these newly developed measures may not be able to detect improved knowledge with much sensitivity. Participants perceived greater input on research spending and agreed that they should have more input in this area. Perhaps what they did learn about research, from the exercise and each other, or other aspects of their experience, diminished pre‐existing “blind” trust in medical researchers. This warrants further study.

Our follow‐up interviews with participants represent one of the few studies of the longer‐term impact of deliberation. Most deliberators had discussed the priority‐setting task with others after the exercise. Deliberators found hearing and understanding other points of view particularly noteworthy, sometimes expressing surprise at the group's ability to reach agreement. Deliberators not only could hear, understand and respect other points of view, they welcomed it and appreciated it. A few learned about needs in their community and became activated to volunteer and/or advocate. Impact of deliberations on decisions and decision making about resources is an important area for future research.^44^

### 4.1 Limitations

Proportions and associations should be interpreted with caution given sampling did not aim to be statistically representative. When convening face‐to‐face deliberations, random sampling during recruitment does not predictably lead to proportional representation, since obstacles to the willingness and ability to attend group sessions (eg time, transportation, mobility) are not randomly distributed. Instead, we aimed to oversample groups typically underrepresented in both research and policy decision making, and had excellent representation of minority and medically underserved populations. Participants ranged in educational attainment and age, and about half were <200% of the federal poverty level. Women were overrepresented, as is often true in research engaging minority and underserved populations.^45^, ^46^ Finally, exploratory analyses of interviews with deliberators about the impact of participation will need to be validated in future work.

Still, this study incorporated a wide variety of tools for data collection and analysis to measure comprehensively the quality of deliberations about resource allocation. Most measures indicated, from the perspective of deliberators themselves, good quality structures, processes and outcomes.

Our results suggest that structured deliberation using CHAT can produce high‐quality deliberation even on complex prioritization decisions, such as health research spending.

## CONFLICT OF INTEREST

All authors have completed the ICMJE Form for Disclosure of Potential Conflicts of Interest. C. Daniel Meyers reports grants from the Agency for Healthcare Research and Quality and the Patient‐Centered Outcome Research Institute outside the scope of this work. Marion Danis reported that the National Institutes of Health may receive royalties from licensing of the CHAT exercise and that part of these royalties are given to her as a component of her salary. Susan Goold reported that she could receive a portion of royalties for any paid licenses from the University of Michigan Office of Technology Transfer.

## DATA AVAILABILITY

The data that support the findings of this study are available from the corresponding author upon reasonable request.
