# Supplementary material for: Evaluating community deliberations about health research priorities
Source: Health Expect. 2019 Jun 28;22(4):772–84. doi: 10.1111/hex.12931 (PMC6737773; doi:10.1111/hex.12931)
Supplement: Supplementary file 1 [file HEX-22-772-s001.docx]

Appendix.

Contents:

- Appendix Table 1
- Appendix Figure 1
- DECIDERS—Interview guide for evaluating impact on CHAT participants

Appendix Table 1. DECIDERS CHAT content.

| Category | Definition | No coverage | Level 1 | Level 2 | Level 3 |
| --- | --- | --- | --- | --- | --- |
| What causes disease? | Research to find out the root cause of diseases and what makes them more or less likely. This may include where you live, family history, life experiences, or public policies. | Causes and risks of disease are not studied. | (3 markers) Research is done on causes and risks for diseases that affect many people or are very severe. | (5 markers) Research for level 1 plus additional research on causes and risks for diseases that affect fewer people and/or are less severe. | (6 markers) In-depth research on causes of diseases that provides a detailed understanding of a wide variety of diseases. |
| Promote health | Research to find out how to prevent illness, promote health, and encourage screening. This research may include how to help people make healthy choices, how to make workplaces and communities safer and healthier, and how to increase preventive care. | No research on ways to promote health and prevent illness. | (3 markers) Research on basic health promotion and prevention for the general population. This includes research on:  • On helping people make healthy choices,  • Disease prevention  • Simple ways to make jobs and neighborhoods healthier. | (5 markers) Research includes studying  • More complicated ways to make communities, jobs, and other places healthy and safe,  • Ways to promote emotional and mental health  • Promoting physical well-being. | (6 markers) In-depth research related to promoting health and disease prevention that includes:  • Research on effective policies. |
| Communication | Research to find better ways for doctors, health care workers, and patients to communicate and understand each other; providing health information to people in a more organized and straightforward way. | Little or no research on communication with doctors and other health care workers. | (3 markers) Some research on improving patient understanding and communication, including ways to help patients make better decisions about healthcare, and to manage their own health. | (5 markers) More research to improve communication. More funding for research to improve the cultural awareness and sensitivities of doctors and the medical system and to increase trust between patients and doctors. | (6 markers) Funding for larger scale studies to improve communication between patients and health systems (not just doctors). |
| What works better? | Research to compare ways to find, diagnose, treat and manage illness to see what works best for treating diseases while improving and maintaining a good quality of life. | Little or no research compares different ways to detect, diagnose, treat and manage illness to see what works best. | (3 markers) Some research compares different ways to detect, diagnose, treat and manage illness to see what works best. | (5 markers) More research compares different ways to detect, diagnose, treat and manage illness to see what works best. | (6 markers) In-depth research comparing different ways to detect, diagnose, treat and manage illness to see what works best. |
| Health disparities | Research on how to improve the differences in health status by different groups. Sometimes these differences in health can be explained by differences in socioeconomic status, gender, race, ethnicity, sexual orientation, or access to health care. | Little or no research addressing health differences. | (3 markers) Some research to understand health differences. | (5 markers) More research to understand health differences. | (6 markers) A lot of research is done to understand health differences. |
| Families/caregivers | Research on how illness affects those who are not the patient. This includes how to support families and caregivers, and information for caregivers to look after their own health care. | Little or no research done on caregivers, families and communities. | (2 markers) Some research on how family, friends, neighbors, communities, and social support networks affect outcomes. | (4 markers) More research on how family, friends, neighbors, communities, and social support networks affect outcomes. Also includes how illness affects those who are not the patient. | (5 markers) Increased funding for research on how caregivers, families, and communities affect outcomes and ways to limit the amount of time and stress a caregiver spends caring for the patient. |
| Access | Research on how to put healthcare within reach for more people. This includes transportation, cost, hours, and new and different ways to deliver care. | Little or no research on access to care. | (3 markers) Research examines how governmental policies, cost, healthcare workforce and telemedicine affect the availability and convenience of healthcare and patient-centered outcomes. | (5 markers) Research studies also examine how transportation, location of healthcare, and new ways to deliver care affect access and health. | (6 markers) Research studies on pilot projects that improve Access to care are performed in order to look for solutions that can help many people. |
| Improve research | Research on making health research better. This includes better methods, better ways to include patient and public views, better ways to share results, and ways to make research more ethical. | Little or no research on how to make researchers better at what they do. | (2 markers) Some research aims to improve scientific methods. Some research studies how to include patient and public views in research and how to communicate results. Some research on how to make research fairer, less harmful, and how to improve trust. | (4 markers) Research is done to support for more community-based research. Researchers study how to improve the experience of research for patients, families, and communities and how to improve research ethics. | (5 markers) In-depth research studies to improve research are performed in order to implement solutions broadly. |
| Health care quality | Research to understand patient needs and wishes and how to improve their quality of care. This includes finding new ways to deliver care or use technology, understand patient needs and wishes or improve patient satisfaction. | Little or no research to understand patient needs and wishes and how to improve their quality of life. | (3 markers) Some research to understand patient needs and wishes and how to improve their quality of life. | (5 markers) More research to understand patient needs and wishes and how to improve their quality of life. | (6 markers) In-depth research to understand patient needs and wishes and how to improve their quality of life. |
| Aging | Research on how to improve care for the aging population. This includes knowing more about diseases dealing with aging, improving care to increase the quality of life of the elderly. | Little or no research on how to improve care for the aging population. | (3 markers) Some research on how to improve care for the aging population. | (5 markers) More research on how to improve care for the aging population. | (6 markers) In-depth research on how to improve care for the aging population. |
| Child health | Research on how to improve care for pregnant women, babies, children and adolescents. This includes understanding differences in populations of children, social determinants specific to that age. | Little to no research on how to improve care for pregnant women, babies, children and adolescents. | (3 markers) Some research on how to improve care for pregnant women, babies, children and adolescents. | (5 markers) More research on how to improve care for pregnant women, babies, children and adolescents. | (6 markers) In-depth research on how to improve care for pregnant women, babies, children and adolescents. |
| Health policy | Research on how to improve public health policy and public policy in general. | Little to no research on how to improve health policy. | (2 markers) Some research on how to improve health policy. | (4 markers) More research on how to improve health policy. | (5 markers) In-depth research on how to improve health policy. |
| Healthy environment | Research to find out how and why our environment affects our health. This includes the social environment, environmental exposures, nutrition, and the built environment. | Little to no research to find out what impacts the environment has on health. | (3 markers) Some research to find out what impacts the environment has on health. | (5 markers) More research to find out what impacts the environment has on health. | (6 markers) In-depth research to find out what impacts the environment has on health. |
| Culture and beliefs | Research to find better ways for providers to be culturally aware, sensitive, and knowledgeable about the differences in ideas about health in people of all cultures, backgrounds, and beliefs. This may include research on language and cultural awareness and sensitivity of providers. | Little to no research in this area. | (2 markers) Some research in this area. | (4 markers) More research in this area. | (5 markers) In-depth research in this area. |
| Mental health | Research about how to improve the well-being of people with emotional or mental illness, or substance abuse issues. | Little or research for individuals of all ages with mental, emotional, or physical problems. | (3 markers) Research into some of the basics for individuals of all ages with mental, emotional, or physical problems. This addresses only the broadest issues and populations. | (5 markers) Research for individuals of all ages with mental, emotional, or physical problems that is starting to be more targeted for different populations and ages. The research findings, however, are very slow. | (6 markers) Research for individuals of all ages with mental, emotional, or physical problems that is targeted. |
| Multiple conditions | Research to look at better ways to treat patients with two or more health problems. This includes how to help these patients manage their care to improve their quality of life. | Little or no research for improving the care of those with multiple conditions. | (3 markers) How to treat and manage multiple health problems that are often found together. | (5 markers) Research studies how to treat and manage multiple health problems less often found together. | (6 markers) Increased research on improving care for those patients with multiple conditions. |

**Appendix Figure 1.** Map of focus group locations and medically underserved areas and populations in Michigan.


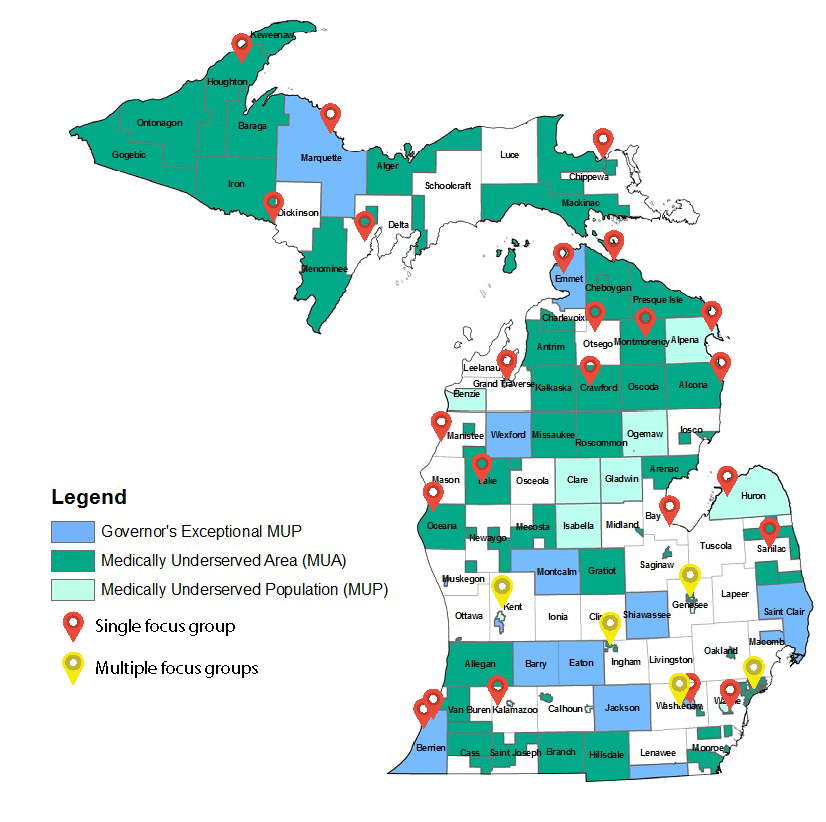


**DECIDERS—Interview guide for evaluating impact on CHAT participants**

Hello,

My name is _______________ and I am a member of a research team at the University of Michigan working on a project called DECIDERS. Sometime last two years, you participated in the DECIDERS project by playing a game called CHAT with fellow community members. You played the game on an Ipad. The game looked like a pie with sixteen different wedges, and you set priorities for health research as an individual and member of a larger group. After playing CHAT, you filled out a form indicating that you would be interested in participating in a follow up interview for a $10 gift card. Are you still interested?

If no 🡪 Ok, thank you for your time.

If yes 🡪 Ok, great! Would you have time to do the interview now (10 to 15 minutes)?

If no 🡪 Is there a better time to reach you?

If yes 🡪 Alright. Today, I would like to ask you a few questions about your experiences on participation in CHAT and the results.

[IMPACT ON PARTICIPATION]

Interview Questions: We would like to ask you some questions on your experience with CHAT

1. What do you remember about playing CHAT?
2. What was it like?
3. What do you remember most about your involvement?
4. Have you ever participated in anything like CHAT before or since?
5. How did your experience affect you?
   1. Have you thought about it?
   2. Have you talked to anyone about it?
      1. if so, who?
      2. Can you tell me a little about that conversation?
6. Have you done anything else since your participation in the CHAT?
   1. Have you looked for additional information about medical science or health research after playing CHAT?
   2. Have you looked for opportunities to get involved in your community ?
   3. Do you think your involvement in this project has made you more or less open to participating in future research studies or has it made no difference at all?
   4. Is there anything else that you think you will or might do differently as a result of your participation in CHAT?
7. What have you read, heard, or experienced that made you think about the CHAT session?
8. What other decisions do you think could benefit from using the sort of group deliberation that you did about health research?

[IMPACT ON RESULTS]

Now I am going to ask you a few question about the results of the project. Did you receive the (email/letter/one-pager/booklet)?

1. Do you remember receiving results for the CHAT project?

(If no – ask if we can resend and the best method. Can we follow up after they receive results?)

(If Yes continue with Question 2)

1. Do you have any questions?
2. What stood out in these results as most significant or important?
3. Did anything surprise you?
4. Have you used or acted on the results in any way? (If so, how)?
   1. Did you talk to anybody?
   2. What do you plan to do?
   3. What is one thing you might do as a result of reading these results?
   4. How do you think the results should be or could be used?
